# Neuromorphic Atomic Switch Networks

**DOI:** 10.1371/journal.pone.0042772

**Published:** 2012-08-06

**Authors:** Audrius V. Avizienis, Henry O. Sillin, Cristina Martin-Olmos, Hsien Hang Shieh, Masakazu Aono, Adam Z. Stieg, James K. Gimzewski

**Affiliations:** 1 Department of Chemistry and Biochemistry, University of California Los Angeles, Los Angeles, California, United States of America; 2 California NanoSystems Institute, University of California Los Angeles, Los Angeles, California, United States of America; 3 World Premier International Center for Materials Nanoarchitectonics, National Institute for Materials Science, Tsukuba, Ibaraki, Japan; Wake Forest School of Medicine, United States of America

## Abstract

Efforts to emulate the formidable information processing capabilities of the brain through neuromorphic engineering have been bolstered by recent progress in the fabrication of nonlinear, nanoscale circuit elements that exhibit synapse-like operational characteristics. However, conventional fabrication techniques are unable to efficiently generate structures with the highly complex interconnectivity found in biological neuronal networks. Here we demonstrate the physical realization of a self-assembled neuromorphic device which implements basic concepts of systems neuroscience through a hardware-based platform comprised of over a billion interconnected atomic-switch inorganic synapses embedded in a complex network of silver nanowires. Observations of network activation and passive harmonic generation demonstrate a collective response to input stimulus in agreement with recent theoretical predictions. Further, emergent behaviors unique to the complex network of atomic switches and akin to brain function are observed, namely spatially distributed memory, recurrent dynamics and the activation of feedforward subnetworks. These devices display the functional characteristics required for implementing unconventional, biologically and neurally inspired computational methodologies in a synthetic experimental system.

## Introduction

The human brain is the most powerful information processor known to man. Although the activity of individual neurons occurs orders of magnitude slower (ms) than the clock speeds of modern microprocessors (ns), the human brain can greatly outperform CMOS computers in a variety of tasks such as image recognition, especially in extracting semantic content from limited or distorted information, when images are presented at drastically reduced resolutions [1]–[4]. These capabilities are thought to be the result of both serial and parallel interactions across a hierarchy of brain regions in a complex, recurrent network, where connections between neurons often lead to feedback loops [5]–[7]. Recent research in systems neuroscience has developed models to explain this combination of rapid and complex processing which view the brain as a large network containing many recurrent loops with both excitatory and inhibitory connections, within which feedforward sub-networks are embedded for fast signal propagation [6], [8], [9].

In the brain, these excitatory/inhibitory connections between neurons, known as synapses, are nonlinear electroionic junctions whose conductivity changes in response to electrical and chemical signals. The relative timing of signals arriving from either side of the synaptic terminals, as well as larger-scale spatiotemporal patterns of network activity during these events, strongly influence the resultant change in synaptic strength, or plasticity [10], [11], a property postulated as the mechanistic basis for memory and learning [12]. Recently, nanoscale electroionic circuit elements known as atomic switches [13] have been shown to exhibit input-dependent memory behaviors similar to short-term plasticity and long-term potentiation in neuronal synapses, where the time constant for conductance decay to the high resistance OFF state depends on the strength and timing of applied voltage pulses [14]. This tendency to equilibrate produces short- and long-term memory behaviors that enable atomic switches to function as “inorganic synapses” [15].

We present a detailed analysis regarding the consequences of coupling many atomic switches together in a highly interconnected, recurrent structure to create an operational neuromorphic device that self-assembles into a functional state. The motivation for building complex network-based computing devices extends beyond an interest in understanding and emulating brain function. Alongside efforts to reduce the dimensions of circuit elements while increasing their integration, the wiring of interconnects has become the limiting factor in both design and performance of electronic devices [16]. Wire delays are significantly slower than transistor switching speeds, producing a situation where more logic gates can be fabricated on a chip than are able to communicate in one processor cycle [17]. This communication bottleneck can be addressed theoretically through the use of different network topologies, varying the number and type of interconnections. Complex nanowire networks are relatively simple to fabricate using self-assembly and would therefore be ideal wiring architectures, provided that they are useful.

Previously we reported an operational regime near the “edge of chaos” in similar network devices, as characterized by power law scaling of temporal metastability, avalanche dynamics and criticality [18] reminiscent of electrical activity in biological neural systems [19], [20]. In such a state, the system is highly correlated and theoretically achieves maximum efficiency of information transfer while retaining a fading memory of prior states. These results indicate a potential capacity for efficient information processing, thereby surmounting problems associated with wire delays and interconnect structures. The distributed nature of the atomic switch array's dynamics makes it a candidate platform for efficient kernel design in the emerging field of “Reservoir Computation” (RC) [21]. The fact that RC does not require subtle control of internal network dynamics and is therefore simpler to execute, makes it an appealing route to begin using neuromorphic devices to perform computational tasks. Complex network architectures generated through self-assembly of functional nanoscale elements, like those described here, offer the benefits of scalability and ease of fabrication combined with control of distributed nonlinear dynamics that may represent the architectural basis of a new computational paradigm.

## Results

Atomic switch network devices were characterized using a range of potentiostatic inputs, including constant and ramped DC as well as sinusoidal AC signals. These complex atomic switch networks are shown to exhibit various nonlinear behaviors, depending on the magnitude and timing of both present and prior input signals. Behaviors include both weak (continuous I–V loop hysteresis) and strong (discrete threshold switching) memristance as well as nonlinear frequency response (higher harmonic generation) and persistent fluctuations in conductivity under constant bias (recurrent connectivity); results which were found to agree with a recent theoretical study of current flow in memristor networks [22]. Operation of the device using pulsed voltage stimulation produced network-specific emergent behaviors, as spatially localized conductive channels akin to feedforward subnetworks were formed within the embedding recurrent network. While there are significant differences between these atomic switch networks and biological neural networks (NNs), we demonstrate the physical implementation of high-level NN features in an inorganic structure, including bottom-up self-assembly that is reminiscent of neuronal growth in the brain [23], nonlinear input-dependent conductance response which strongly resembles the function of biological synapses [11], [12], and emergent properties considered fundamental to brain function - recurrent dynamics which gives rise to large persistent, correlated network responses and the activation of feedforward subnetworks [8], [9], [24]–[27].

### Atomic switches, complex networks and neuromorphic hardware

Previous reports on the synapse-like properties of single atomic switches have demonstrated features similar to short-term plasticity and long-term potentiation, where applied bias voltage produced a junction conductance dependent on the history of stimulation (pulse frequency, length) [14]. Individual atomic switches exhibit time-dependent nonlinear conductance due to several related mechanisms: (1) bias induced Ag^+^ migration, (2) electrochemical redox reactions involving Ag^+^/Ag^0^ to produce metallic filaments, and (3) an associated non-equilibrium α/β-Ag_2_S phase transition [28], which all compete with thermodynamically driven stochastic renormalization to the equilibrium OFF state. Though atomic switches can be configured to operate in an essentially nonvolatile manner similar to memristors—two-terminal circuit elements whose resistance depends on the history of charge passed through them [29]—their volatility indicates that they are more properly classified as “memristive systems” [30], [31].

These mechanisms collectively produce the memristive switching and synaptic memory functions exhibited by a single atomic switch. Specifically, ‘weak’ memristance resulting from redistribution of Ag^+^ dopant cations across the insulator leads to ‘strong’ memristance characterized by abrupt switching through metallic filaments formed once the Ag^+^ cations reach the cathode and are reduced to metallic silver [13]. TEM studies have shown that the metallic silver filaments formed during switching are surrounded by a sheath of β-Ag_2_S, a conductive phase of silver sulfide normally unstable below 170°C [28], possessing a body-centered cubic structure with sulfide anions forming channels in which silver cations are delocalized, highly mobile and dynamically correlated [32], [33]. This non-equilibrium phase transition is attributed to a relaxation of strain induced by lattice mismatch between Ag^0^ and α-Ag_2_S, the electrically insulating room temperature phase [34]. In the absence of applied bias, thermodynamic pressures return the Ag_2_S to its room-T α-phase, which drives the dissolution of the Ag^o^ filaments and turns the atomic switch OFF at a rate dependent on the history of applied bias, producing the observed memory effects.

A great deal of effort has been put towards building biologically inspired computational hardware [35]–[42], though matching the complexity of the brain in a usable electronic device presents an exceedingly difficult engineering challenge. Fabrication requirements force design concessions, such as approximating the complex, recurrent connectivity between neurons by a simpler network geometry. The amenability of crossbar structures to conventional fabrication techniques has led to their use in neuromorphic hardware, with pre- and post-synaptic CMOS neurons connected by memristive elements at the crosspoints [43]. This is an ideal hardware implementation of a 3-layer neural network model [44], where input and output neurons are connected by a synaptic “hidden layer” of variable strength, and is also a promising platform for building dense, fast solid-state memory devices [45]. However, the structural simplicity of the crossbar architecture is both a strength, enabling independent control of each synaptic element, and a weakness, since the well-defined grid lacks complex structures with the recurrent connections believed to be essential to brain function [6], [25]. While it is possible to program these features into a software model implemented on neuromorphic hardware, the physical existence of these complex structures may be essential to successfully generate the requisite spatiotemporal interactions between multiple signals simultaneously traveling through the network [11], [46].

### Device fabrication and characterization

**Figure 1:**
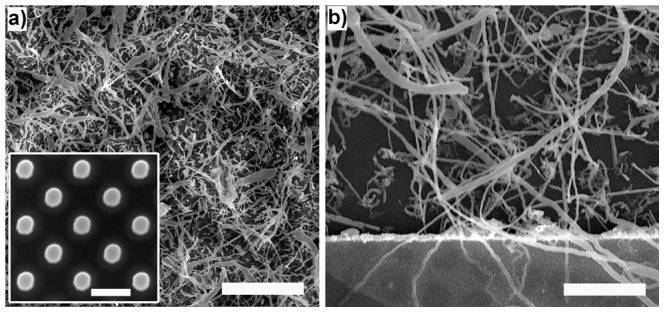
Device Fabrication. (a) SEM image of complex Ag networks (scale bar = 10 µm) produced by reaction of aqueous AgNO_3_ (50 mM) with (inset) lithographically patterned Cu seed posts (scale bar = 1 µm). (b) High resolution image of the functionalized Ag network at the device electrode interface (Pt) showing wire widths ranging from 100 nm to 3 µm (average <1 µm) and lengths extending from a few microns to almost a millimeter (scale bar = 700 nm).

Based on the view that recurrent connectivity is essential to brain-like function, we have built, characterized and operated devices using massively interconnected (10^9^ junctions/cm^2^ according to analysis of SEM images), silver nanowire networks functionalized with interfacial Ag|Ag_2_S|Ag atomic switches. These nanowire networks were prepared through self-assembly without pre-patterning of the network topology using the electroless deposition of Ag from Cu inside the SU-8 reaction well of an I/O device platform [18], [47]. Specifically, spontaneous oxidization of metallic copper through reaction with dilute aqueous solutions of AgNO_3_ produces a metallic silver structures with variable morphologies depending on the concentration of Ag^+^ and distribution of Cu [48]–[50]. Dendritic silver nanowires with minimum feature sizes <100 nm seen in Figure 1b were produced by using lithographically patterned Cu posts shown in the inset of Figure 1a. Control over the size and distribution of Cu seeds increased device yield by ensuring the formation of conductive pathways between the Pt device I/O electrodes as seen in Figure 1b. Ag|Ag_2_S|Ag interfaces were formed spontaneously within the network during gas phase sulfurization [51]. Following optimization of fabrication protocols, a total of 96 networks were used for the device characterization described below.

**Figure 2:**
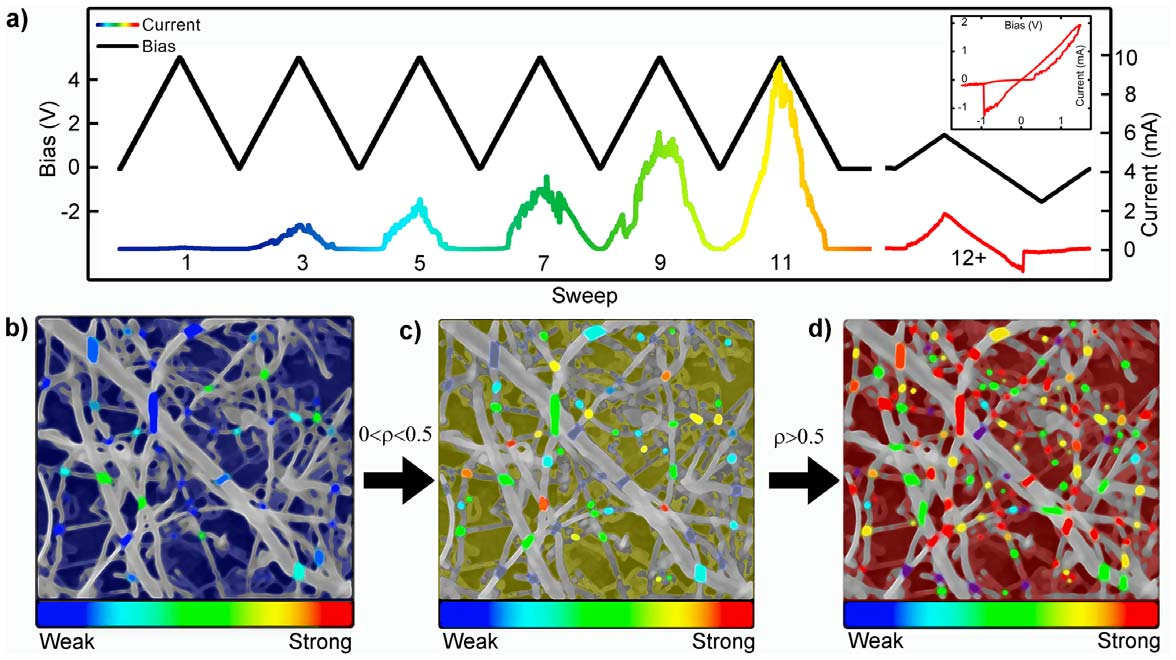
Network Activation - memristive behavior. (a) Representative example of initial bias sweeps (0–5 V sweep at 1 V/s) applied to a pristine device which steadily activate higher percentages of atomic switches, resulting in increased current. After 11 sweeps, the device resistance decreases from ∼10 MΩ to ∼500 Ω. Subsequent ±1.5 V bipolar sweeps result in repeatable pinched hysteresis behavior (inset: R_OFF_ = 25 kΩ, R_ON_ = 800 Ω), and bistable switching. (b–d) Schematic representation of the mechanism producing the I–V characteristics shown in (a). The network initially consists of weakly memristive junctions and ohmic contacts (b). Continued application of unipolar bias voltage (c) drives the dissolution of silver into silver sulfide, increasing the number of memristive elements, while cation migration across extant memristive junctions leads to filament formation and the onset of hard switching behavior. (d) After the proportion of strong memristors exceeds the percolation threshold (ρ>0.5), the network functions reliably in the hard switching regime.

Theoretical analysis of current flow in memristor networks during bias voltage sweeps indicated the possibility of a phase transition in device behavior from ‘weak’ to ‘strong’ memristive regimes [22]. Initial voltage sweeps of these network devices (Figure 2a and S1) typically demonstrated smooth, pinched hysteresis loops characteristic of weakly memristive systems followed by an abrupt, nearly discontinuous jump to a distinct, high conductance ON state occurs at an activation bias voltage (V_a_). This behavior represents activation of the network and is shown as an illustrative example of a network device undergoing a behavioral phase transition similar to the bias-driven forming step required to activate single resistive switches. Following network activation, devices subjected to repeated bias sweeping generally exhibit robust, strong memristive behavior, typified by hard switching (inset). Robust switching over 10,000 cycles was demonstrated at an operational threshold voltage (V_t_) of reduced magnitude (Figure S2) as compared to the formation bias voltage, a general phenomenon in resistive switches [52]. While the specific magnitude of V_a_ and V_t_ differ significantly between devices due to inherent variability in the solution-phase methods used to fabricate them, the qualitative transition from weak to strong memristive behavior was observed regularly, consistent with theoretical predictions [22].

Similar to the electroforming step usually required to activate single atomic switches and memristors [52], the observed transition from weak to strong memristive behavior is assigned to two related mechanisms. In poorly conducting regions comprised mainly of Ag_2_S, anodic silver dissolves into and travels across the electrically insulating sulfide as Ag^+^, decreasing resistance and producing a weakly memristive effect. In regions of higher Ag^+^ dopant concentrations, mobile cations reach the cathode and are reduced to Ag^0^, creating metallic filaments across the insulator that cause an abrupt change to an ON state with a sharp increase in conductance at V_t_ associated with the electrochemical process of filament formation. At the network level, the bias-induced creation of additional memristive junctions and filament formation across existing ones combine to produce the theoretically predicted transition of network I–V behavior to a strongly memristive phase (schematically illustrated in Figure 2b–d) as the proportion of switching elements in the network exceeds the percolation threshold (50%) [22]. Having undergone this transition, the continuously swept network operates as a hard switching memristor shown in Figure 2a (inset). All further data presented was acquired from devices following activation.

### Network-specific properties

While weak and strong memristive behavior can be exhibited by single resistive switches, the most interesting features of this complex atomic switch device are its network-specific properties. In order to confirm that the entire network was involved in processing the input signals, devices were imaged using an IR camera with 20 mK sensitivity to track Joule heating from current flow during slow bias sweeps. The IR images revealed power dissipation occurring across the network, indicating that the phase change in network I–V behavior was not attributable to the formation of a single maximum conductivity pathway of switches arranged in series between the active electrodes [18]. The distribution of activity indicates that the observed I–V characteristics are due to the sum of parallel current flow, meaning that network structure and connectivity are actively influencing device function.

**Figure 3:**
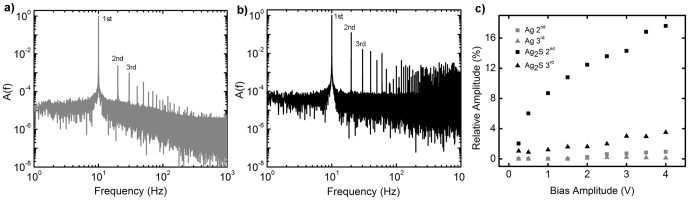
Frequency Response – distributed conductance. (a) Amplitude spectrum from a Fourier transform of a control device's response to a 2 V, 10 Hz sinusoidal input signal compared to (b) that of a functionalized device which shows enhanced overtones of the input signal with respect to (a). (c) Plot of 2^nd^ and 3^rd^ harmonic generation in current response as a function of bias voltage in both functional (black) and control (gray) networks. Harmonic magnitudes are represented as percentage of the fundamental for a 10 Hz sinusoidal input signal.

As recent theoretical models predict passive generation of second harmonics in both singular memristors and in random networks, the distribution of switch function throughout the network was examined through analysis of the device's frequency response [22], [53]. Simulation of current flow in memristor networks indicate that 2^nd^ harmonic generation will occur under an applied sinusoidal voltage in networks whose percentage of hard switching junctions exceeds the percolation threshold [22]. Further, the relative magnitude of higher harmonics is predicted to increase with the relative number of hard switching junctions. Following activation, device response to a 10 Hz sinusoidal voltage signal varying in strength from 250 mV to 4 V shows a large increase in higher frequency components after functionalization (Figure 3b). The proportion of higher harmonics generated increases with signal amplitude (Figure 3c), with the largest increase occurring between 250 and 500 mV. A larger degree of higher harmonic generation is consistent with an increased number of memristive junctions operating in the hard switching regime above V_t_ (∼0.5 V). Both the distributed power dissipation [18] and harmonic generation are characteristic of activity distributed throughout the network.

Having characterized atomic switch operation in an interconnected complex network, we examined the device for emergent behaviors specific to its brain-like recurrent structure. Structurally, the atomic switch network is recurrent in the sense that there exist pathways such that electrical signals produced at one junction may lead to (delayed) feedback at the same junction. Here we present experimental evidence of spatially distributed and correlated network dynamics, which are attributed to such recurrent connectivity. These recurrent dynamics are presented as an emergent property of the atomic switch network.

**Figure 4:**
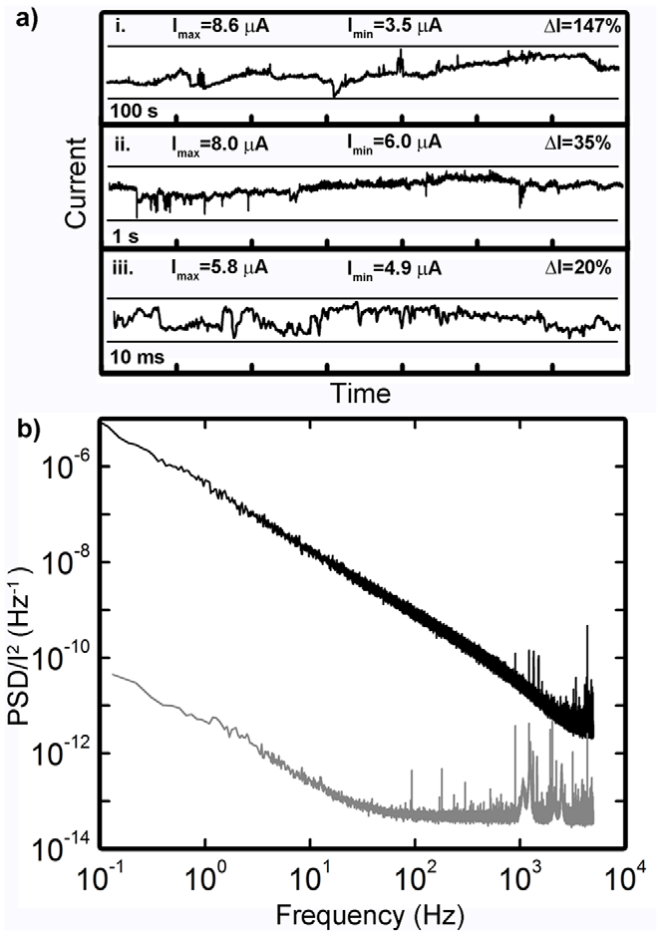
DC Response – recurrent dynamics. (a) Time traces of current response to 1 V DC bias show large current increases and decreases at all time scales around a mean of 5.81 µA (172 kΩ); shorter time traces (ii–iii) are subsets of (i). Representative device parameters: R_OFF_>10 MΩ, R_ON_<20 kΩ, V_T_ = 3 V during activation (b) Fourier transforms of DC bias response for Ag control (grey) and functionalized Ag-Ag_2_S (black) networks. The power spectrum of the functionalized network displays 1/f^β^ power law scaling (β = 1.34).

Applying a constant 1 V DC bias (Figure 4a) produced persistent, bidirectional fluctuations—both increases and decreases—in network conductivity of large magnitudes (∼20–150%) over a range of time scales (seconds-hours). In the absence of recurrent structures within the network, the filamentary mechanism of an atomic switch implies that conductivity would increase monotonically under constant DC bias. The applied voltage leads to the thickening of filaments until the potential drop across the junctions is insufficient to reduce more silver cations [13]. However, large bidirectional fluctuations (ΔI greater than 100% on the scale of hours) in the current response persisted for several days under constant applied voltage, demonstrating that the complex network connectivity inherently resists localized positive feedback that would lead to the serial formation of a single, dominant high conductivity pathway between electrodes. Rather, recurrent loops in the network create complex couplings between switches, resulting in network dynamics that do not converge to a steady state even under constant bias. A single switch turning ON does not simply lead to an increased potential drop across the next junction in a serial chain, but redistributes voltage across many recurrent connections that can ultimately produce a net decrease in network conductivity. This behavior represents a network-scale analog of defect-defect interactions that have been observed to produce current fluctuations in metal nanobridges [54]. The nanoscale switch filaments couple these interactions with electrochemical redox processes, leading to significant changes in the conductivity state of the entire network.

These fluctuations are of a magnitude significantly greater than what can be considered noise. An internal control experiment compared Fourier transformed current responses (Figure 4b) of the devices to constant voltage before and after functionalization. The formation of atomic switch junctions expands the degree of correlation in current fluctuations, producing 1/f-like behavior across the entire sampled range, far exceeding that of control devices (unsulfurized silver network, grey line in Figure 4b), which flattens to white noise and some high energy, high frequency fluctuations attributed to arcing between neighboring wires. Functionalization with atomic switch elements increases the influence of past events on the present state of the network, in accordance with their memristive characteristics [14], [15], [55]. This results in an expanded degree of correlation in the measured frequency response. Similar 1/f spectra have been observed along with current fluctuations in other resistive switching systems, exhibiting relative resistance changes ΔR/R ranging from <0.002 for metallic filaments to an experimental and theoretical maximum of 0.5 in the semiconducting high resistance OFF state [56]. The network device of Figure 4 is operating in an intermediate state with an average resistance of 172 kΩ (compared to R_OFF_>10 MΩ) and fluctuations of ΔR/R∼1. In order to produce relative resistance changes of such high magnitude, switching events within the network must be correlated. While stochastic processes may be involved in the correlation of these fluctuations [55], [57], their magnitude and persistence is an emergent feature of recurrent connectivity in the device architecture that has not been observed in simpler atomic switch geometries.

**Figure 5:**
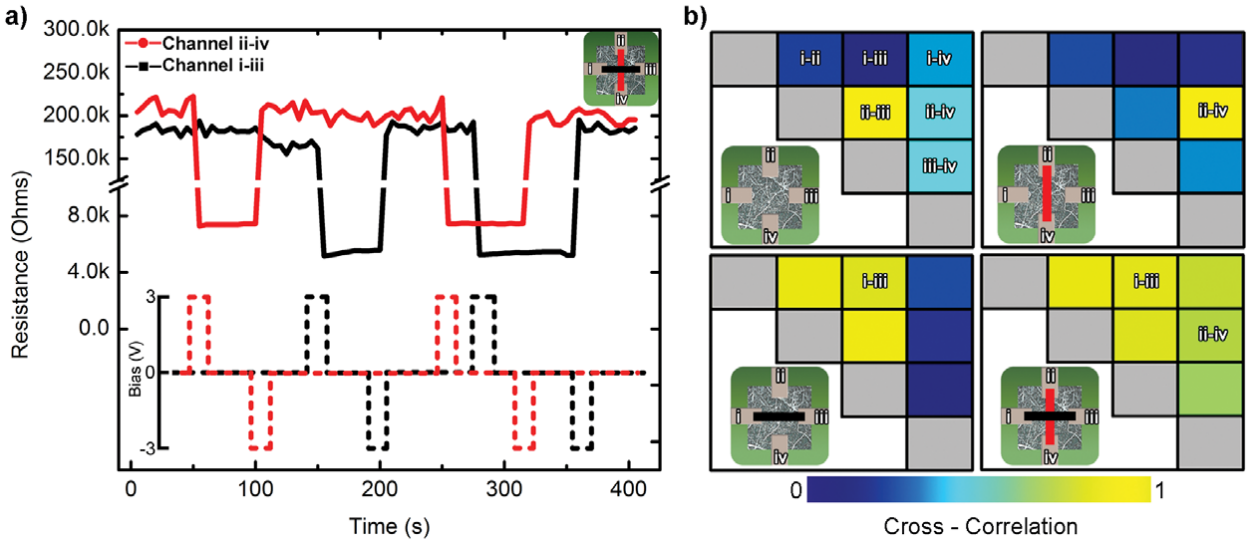
Distributed Memory Storage from Network-scale Switching. (a) The device operates as a 2-bit non-volatile memory device. The resistance states across two channels (i–iii and ii–iv) are monitored. ON/OFF switching of each channel is induced using super-threshold pulses (3 V, 1 s in duration); the threshold voltages for each channel are ∼1.5 V. The resistances are measured every 5 s with a sub-threshold 200 mV, 100 ms pulses. (b) Although the device operates with a four state output (both channels ON, 1 ON/1 OFF, etc), the network's internal configurations show diverse correlated patterns, from no correlation (blue) to total correlation (yellow). The figure shows correlation coefficients of channel resistances for all 6 pairwise electrode combinations. The correlation coefficients are calculated during each of the 4 network switching configurations; the black and red bars (insets) show the channels that are ON in the switching state.

Inside the generally recurrent structure of the brain's neural network, there is evidence for the existence of feedforward subnetworks utilized for the fast propagation of certain signals [24]. In this device, persistent fluctuations in current under constant DC bias are produced by the recurrent network architecture, creating operational dynamics that resist the feedfoward activation of serial chains of switches. However, by altering the form of the input signal, we were able to independently operate conductance channels between different pairs of electrodes within the same device. The application of a single, large voltage pulse (±3 V, 1 s) selectively switched connections between electrode pairs ON and OFF (Figure 5a) with a R_ON_/R_OFF_ ratio greater than 30. In the example shown, the conductive paths between the two channels overlap spatially, yet are switched independently, indicating that local sub-regions of the network can transition to distinct operational modes despite being embedded within a highly interconnected, largely metallic structure. This is analogous to the presence of feedforward subnetworks within the recurrent architecture of the cortex. Single pulses of sufficient magnitude overwhelm the recurrent dynamics and induce feedforward activation of local sub-regions along a path connecting the involved pair of electrodes without significantly altering the conductivity of other spatially intertwined channels within the same nanowire network.

The degree to which pulse-mode channel creation influences overall network connectivity can be visualized in electrode resistance cross-correlation matrices (Figure 5b). In this case, net electrode resistance is calculated from the pair-wise resistances to be a representative measure of the overall connectivity of a given electrode to the network. The correlation strength (denoted by color) represents the degree to which a pair of net electrode resistances fluctuate in unison, interpreted as a measure of the number of shared network sub-regions connected to both electrodes (Supporting Information). Correlation strength increases strongly between electrodes connected by an ON channel, and decreases again when the channel is switched OFF, with a varying degree of influence on electrodes not directly involved in the switching. This implies that spatially central regions of the network can be selectively associated with particular pairs of electrodes without globally increasing the network connectivity. However, when conductive channels exist between all four electrodes, the overall magnitude of correlation in the network is correspondingly large, as fluctuations are spread evenly throughout the increasingly metallic network. This simple example of the interaction between local and global operational characteristics is a promising indicator of the possibility for the creation of a brain-like hierarchy of distinct functional regions within a single network where the functional connectivity of the network itself is both dynamic and self-organized [58].

## Discussion

Using a simple, two-step fabrication procedure combining top-down and bottom-up fabrication techniques, we have created functional neuromorphic devices based on a self-assembled, complex network architecture. We describe these atomic switch networks as neuromorphic not only in that the massively interconnected, dendritic features observed in biological neural networks inspired the device architecture, but also due to several important network scale properties reported here. The devices demonstrate weak and strong memristive behaviors, as well as higher harmonic generation, confirming theoretical predictions on current flow through memristor networks. Previously unreported emergent behaviors specific to the complex architecture were observed as persistent bidirectional fluctuations of the current in response to constant applied voltage and the pulse-based feedforward switching ON and OFF of localized conductance channels within the highly interconnected network. Despite lacking the brain's rich assortment of neurotransmitter systems, with distinct excitatory and inhibitory neurons, the complex network of atomic switches produces multiple behaviors from a single basic unit through a capacity for localizing function in subnetworks inside a structure correlated by the nonlinear memory response of individual atomic switches. This diversity of function indicates the device's potential as a universal approximator of dynamical systems [59], with possible applications in physically implementing unconventional computational strategies [21] and as an inorganic experimental platform for the investigation of systems neuroscientific theories of biological brain function.

## Materials and Methods

### Substrate Fabrication

Electrodes were patterned on the surface of a Si wafer (525 µm thickness; p-type; 100 mm diameter; 500 nm thermal oxide) by photolithography. A Cr/Pt (15/150 nm) bilayer was deposited using e-beam evaporation. Subsequently, microfluidic reaction wells were patterned from a thick layer of SU-8 (approx. 500 µm) deposited by spin coating. The resist was UV exposed with a dose of 1200 mJ/cm^2^ followed by a post-exposure bake beginning at 65°C and ramping up to 95°C before cooling to room temperature at 1°C/min. The SU-8 was developed by immersion in PGMEA (Propylene Glycol Methyl Ether Acetate). Fully developed wafers were rinsed with isopropanol and hard baked at 130°C on a hotplate in N_2_ atmosphere to increase SU-8 resistance to high temperatures.

### Network Synthesis and Functionalization

Electroless deposition of Ag from Cu was performed by pipetting aqueous AgNO_3_ (Fischer, 99.98%) at concentrations ranging from 0.1–100 mM into microfluidic cells containing Cu seed posts, leading to a spontaneous reaction between Ag^+^ and Cu. Optimal conditions were achieved with Cu posts ranging from 0.25–4 µm in diameter at pitches of 0.5–4 µm reacted with 50 mM AgNO_3_, sulfurized at 130°C for 10 minutes under N_2_ flow at atmospheric pressure. The silver networks self-assembled during this processes, and were then functionalized by reaction with sulfur (Sigma-Aldrich, 99.5%) in a Pyrex tube. The sulfur was melted in an evaporation boat at 130°C and delivered to the substrate by N_2_ flow.

### Measurement Apparatus

Electrical characterization of the devices was conducted using four Pt electrodes positioned around the edges of the Ag network. Current-voltage spectroscopy was conducted using a bipotentiostat (Pine Instruments model AFCBP1) in conjunction with a DAQ module (National Instruments USB 6259) at a sample rate of 10 kHz. Measurements were performed in a two-electrode configuration. Multi-channel resistance measurements were obtained using a multiplexed (National Instruments PXI 1073) SMU (National Instruments PXI 4130). The entire I/O system was housed in a Faraday cage and mounted on a vibration isolation table (TMC). Devices were characterized after each stage of the fabrication cycle. Subsequent data analyses were carried out using MATLAB 2010b (MathWorks) and Origin 8.1 (OriginLab Corporation).

### Network Resistance Correlations

The full dataset used in Figure 5b contained resistance data from all 6 combinations of the 4 electrodes in a device (for clarity, only 2 combinations are shown in Figure 5a). The network resistance of each electrode was calculated as the parallel resistances to the other 3 electrodes. The dataset was parsed into the appropriate subsets (A on and B off, etc.) and the MATLAB function corrcoef() was used to calculate the correlation coefficients for the different configurations.

## Supporting Information

Figure S1 **Device Activation.** (a) Initial bias sweeps (±7.5 V at 1 V/s) demonstrate weakly memristive behavior with increasing hysteresis magnitude (70% increase in maximum ON/OFF, from 1.12 to 1.92 after 8 sweeps). (b) Bias sweeps from (a) rescaled to include the hard switching (ON/OFF ratio of 14.3, 650% increase from maximum weak ON/OFF) phase transition event at V_a_≈7.5 V.
(TIF)

Figure S2 **Robust Switching.** Operation of a device following the phase transition (activation) exhibiting typical, robust pinched hysteresis/switching. Shown device parameters: sweep rate = 10^3^ V/s (1 kHz), R_ON_ = 1 kΩ, R_OFF_>20 kΩ, V_t_ = 0.5 V.
(TIF)
