# Supplementary figures and images for: Neuromorphic Atomic Switch Networks
Source: PLoS One. 2012 Aug 6;7(8):e42772. doi: 10.1371/journal.pone.0042772 (PMC3412809; doi:10.1371/journal.pone.0042772)

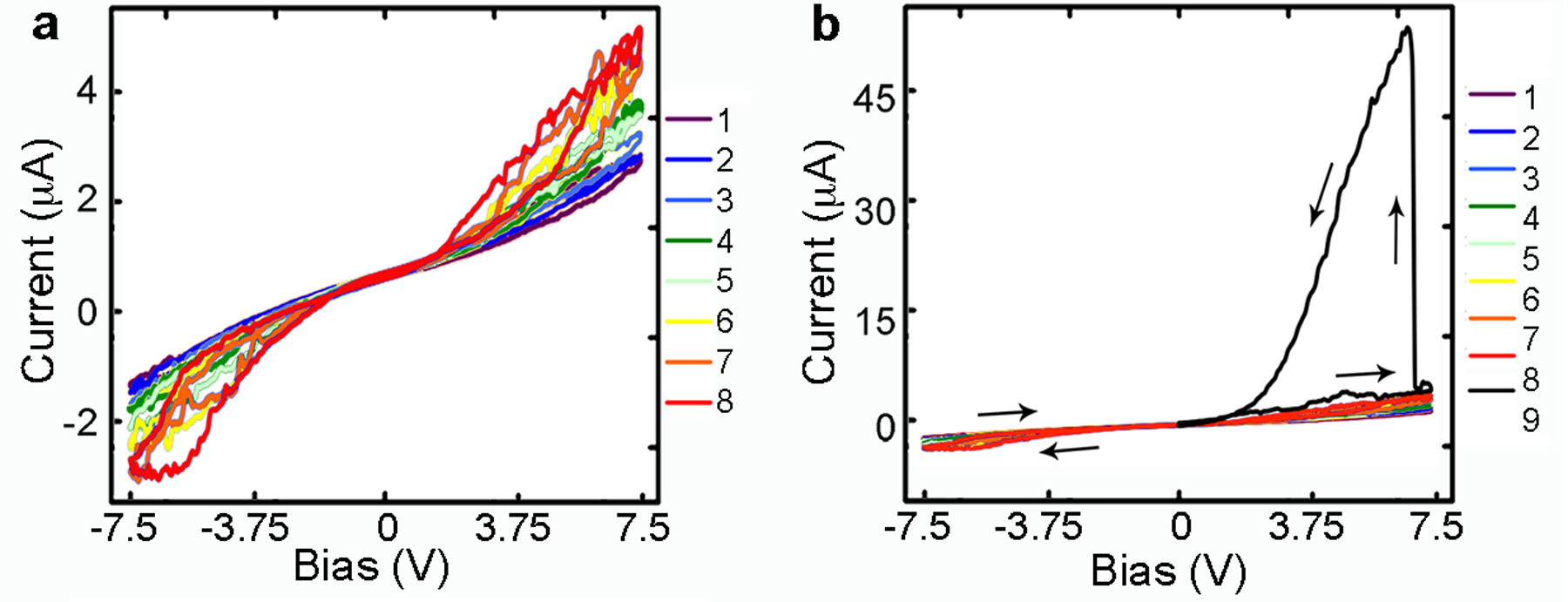

Supplement: Figure S1 — Device Activation. (a) Initial bias sweeps (±7.5 V at 1 V/s) demonstrate weakly memristive behavior with increasing hysteresis magnitude (70% increase in maximum ON/OFF, from 1.12 to 1.92 after 8 sweeps). (b) Bias sweeps from (a) rescaled to include the hard switching (ON/OFF ratio of 14.3, 650% increase from maximum weak ON/OFF) phase transition event at Va≈7.5 V. (TIF) [file pone.0042772.s001.tif]

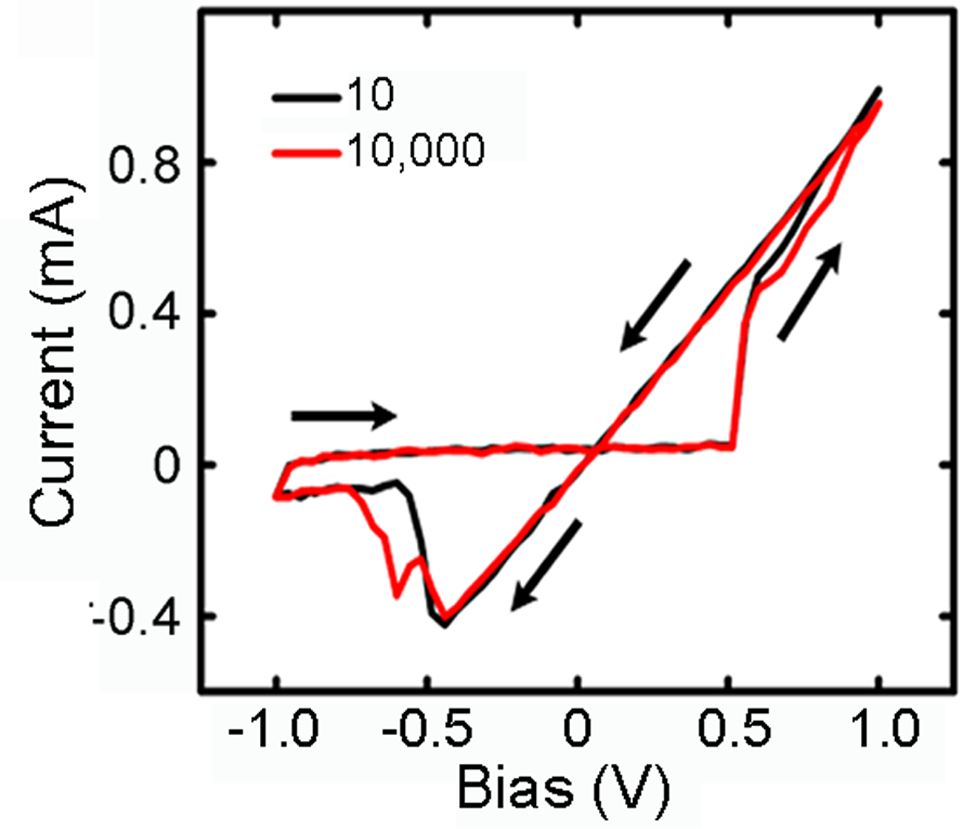

Supplement: Figure S2 — Robust Switching. Operation of a device following the phase transition (activation) exhibiting typical, robust pinched hysteresis/switching. Shown device parameters: sweep rate = 103 V/s (1 kHz), RON = 1 kΩ, ROFF>20 kΩ, Vt = 0.5 V. (TIF) [file pone.0042772.s002.tif]
